# Echocardiographic characteristics of new-onset methamphetamine-associated heart failure: a retrospective cohort study

**DOI:** 10.1186/s44156-026-00133-3

**Published:** 2026-08-24

**Authors:** Joey Chiang, Danelle Hidano, Heather Herren, Christopher T. Longenecker, Mrinal Yadava, Cooper B. Kersey

**Affiliations:** 1 Division of Cardiology, University of Washington School of Medicine, 1959 N.E. Pacific St, Seattle, WA 98195 USA; 2 Institute of Translational Health Sciences, University of Washington, 850 Republican St, Seattle, WA 98109 USA; 3 University of Washington Medical Center, 1959 NE Pacific St, Box 356422, Seattle, WA 98195-6422 USA

**Keywords:** Stimulants, Strain, Non-ischemic cardiomyopathy, Diagnosis

## Abstract

**Background:**

Methamphetamine-associated heart failure (MAHF) is a growing cause of healthcare burden, yet the echocardiographic phenotype at the time of initial diagnosis is poorly characterized. We tested the hypothesis that new-onset MAHF exhibits a distinct echocardiographic phenotype that could aid in early disease identification.

**Methods:**

We conducted a retrospective cohort study comparing echocardiographic parameters in patients with new-onset MAHF versus controls with non-ischemic cardiomyopathy unrelated to methamphetamine use. Patients with MAHF (*n* = 75) were identified by positive urine drug screen, ICD-10 codes for incident heart failure, and confirmed active methamphetamine use at index hospitalization. Controls were matched 1:1 by age, sex, and race. Pre-specified echocardiographic endpoints included left and right ventricular size and function, left ventricular thrombi, valvular dysfunction, and pulmonary artery systolic pressure.

**Results:**

The two groups showed no significant differences in echocardiographic parameters. Left ventricular ejection fraction (28%, 20–36% vs. 25%, 19–35%), left ventricular indexed end-diastolic volume (103 ml/m2, 79-130 ml/m2 vs. 94 ml/m2, 73-118 ml/m2), left atrial size, right ventricular size and function, diastolic parameters, pulmonary artery pressures, and prevalence of ventricular thrombi were all similar between groups.

**Conclusions:**

At the time of initial heart failure diagnosis, MAHF is indistinguishable by traditional echocardiographic parameters from other non-ischemic cardiomyopathies. The findings of this study differ from prior research into the echocardiographic phenotype of MAHF by focusing on the time of initial heart failure diagnosis, presumably when less adverse remodeling has occurred. These negative findings underscore that MAHF is a diagnosis of exclusion and a full work-up to assess for alternative etiologies of cardiomyopathy is paramount.

## Background

Methamphetamine use causes dilated cardiomyopathy through multiple mechanisms: excess sympathetic tone, direct myocyte toxicity, and ultimately myocardial fibrosis [1]. Hospitalizations for methamphetamine-associated heart failure (MAHF) increased from 2008 to 2020, causing substantial healthcare burden [2]. The importance of early detection of MAHF is underscored by the work of Schurer et al. who demonstrated that methamphetamine cessation is associated with less myocardial fibrosis and an improvement in left ventricular ejection fraction, while continued use leads to a progressive decline in systolic function [1]. Early disease detection additionally allows for timely initiation of guideline-directed medical therapy (GDMT) for heart failure (HF) which can yield clinical improvement in MAHF [2]. Prior echocardiographic studies of MAHF have focused on patients with established cardiomyopathy, leaving the echocardiographic phenotype at the time of diagnosis largely undefined. We tested the hypothesis that new-onset MAHF exhibits a distinct echocardiographic phenotype that could aid in early disease identification.

## Methods

We conducted a retrospective cohort study to compare differences in echocardiographic parameters among patients with MAHF versus controls with non-ischemic cardiomyopathy unrelated to methamphetamine use. Informed consent was waived for this retrospective study (IRB protocol 00014632) as it involved review of the existing medical records, posing no more than minimal risk to participants. We identified individuals with MAHF by positive methamphetamine urine drug screen (UDS) and ICD-10 codes for incident HF between 4/1/2021 and 12/31/2022. We adjudicated each case using clinical documentation to confirm active methamphetamine use (*n* = 75) within the week leading up to index hospitalization. Other stimulant use (e.g. cocaine and MDMA) was excluded from our definition of cases or controls. We excluded patients with clear alternative causes of heart failure (ischemic, valvular, congenital); pre-existing cerebrovascular or peripheral vascular disease not implicated in the etiology of the heart failure diagnosis was not excluded. We identified controls using ICD-10 codes for acute heart failure without a history of methamphetamine use and negative UDS. Controls and cases were matched 1:1 for age (+/- 2 years), sex, and race. We compared echocardiographic data from the initial transthoracic echocardiogram performed during the index heart failure hospitalization for both cases and controls. Markers of left and right ventricular size and function, presence of left ventricular thrombi, degree of valvular dysfunction, and pulmonary artery systolic pressure were pre-specified echocardiographic endpoints. Patients with missing diastolic data (due to imaging quality), uninterpretable findings in the setting of atrial fibrillation, or contradictory diastolic parameters had these analyses omitted (missing rate 64%). χ2 or Fisher’s tests were used to test categorical variables; T-tests or Mann-Whitney U tests were used for continuous variables. Because we performed multiple statistical comparisons, a Bonferroni correction was applied.

## Results

MAHF patients had lower BMI, higher rates of using public insurance, and higher rates of housing instability; they also had higher rates of comorbid substance use disorders, schizophrenia, and psychosis when compared to controls (Table 1). There were no other differences in demographic or baseline characteristics between groups.

**Table 1:** Demographics and baseline data

		HF Control (*N* = 75)	MAHF (*N* = 75)	*p*-value	
**Baseline Demographics**					
AGE (YEARS)	Median (IQR)	54.0 (38–62)	54.0 (38–62)	0.912	
Race	White	49 (65.3)	43 (59.7)	0.941	
	Black or African-American	17 (22.7)	18 (25.0)		
	American Indian or Native Alaskan	3 (4.0)	5 (6.9)		
	Asian	4 (5.3)	4 (5.6)		
	Native Hawaiian or Pacific Islander	2 (2.7)	2 (2.8)		
GENDER	Male	59 (78.7)	62 (82.7)	0.535	
	Female	16 (21.3)	13 (17.3)		
Ethnicity	Non-Hispanic	66 (88.0)	65 (90.3)	0.658	
	Hispanic	9 (12.0)	7 (9.7)		
BMI	Median (IQR)	30.2 (24–35)	24.8 (22–30 )	0.002*	
Housing	Unsheltered location (i.e. street, vehicle, tent)	4 (5.3)	41 (54.7)	< 0.001*	
	Short or long term shelter	0	2 (2.7)		
	Assisted living facility (i.e. SNF, LTAC, Respite, Group Home)	1 (1.3)	0		
	Subsidized housing	3 (4.0)	3 (4.0)		
	House or apartment	62 (82.7)	27 (36.0)		
	Staying with friends/relatives	5 (6.7)	2 (2.7)		
Insurance	Medicaid	28 (37.3)	57 (77.0)	< 0.001*	
	Medicare	17 (22.7)	12 (16.2)		
	Private insurance	23 (30.7)	1 (1.4)		
	Uninsured/self pay (not listed)	7 (9.3)	4 (5.4)		
**Medical History**					
**Cardiac**					
Hypertension	No HTN	33 (44.0)	44 (58.7)	0.072	
	HTN	42 (56.0)	31 (41.3)		
Atrial Fibrillation/Flutter	No	61 (81.3)	66 (88.0)	0.257	
	Yes	14 (18.7)	9 (12.0)		
History of Endocarditis	No	74 (98.7)	73 (97.3)	1.000	
	Yes	1 (1.3)	2 (2.7)		
History of Cardiac Arrest	No	75 (100)	74 (98.7)	1.000	
	Yes	0	1 (1.3)		
**Medical**					
Diabetes mellitus	No	61 (81.3)	67 (89.3)	0.166	
	Yes	14 (18.7)	8 (10.7)		
CKD/ESRD	No	59 (78.7)	61 (81.3)	0.683	
	Yes	16 (21.3)	14 (18.7)		
Cerebrovascular accident	No	73 (97.3)	74 (98.7)	1.000	
	Yes	2 ( 2.7)	1 (1.3)		
Vascular disease		No	70 (93.3)	70 (93.3)	1.000
		Yes	5 (6.7)	5 (6.7)	
Obstructive pulmonary disease (i.e.COPD)	No	70 (93.3)	68 (90.7)	0.547	
	Yes	5 (6.7)	7 (9.3)		
HIV/AIDS	No	74 (98.7)	71 (94.7)	0.367	
	Yes	1 (1.3)	4 ( 5.3)		
Any Medical Conditions	No	40 (53.3)	44 (58.7)	0.511	
	Yes	35 (46.7)	31 (41.3)		
**Substance Use (current/prior)**					
Tobacco use disorder	No	64 (85.3)	45 (60.0)	0.001*	
	Yes	11 (14.7)	30 (40.0)		
Alcohol use disorder	No	64 (85.3)	55 (73.3)	0.070	
	Yes	11(14.7)	20 (26.7)		
Opioid use disorder	No	70 (93.3)	50 (66.7)	< 0.001*	
	Yes	5 (6.7)	25 (33.3)		
Any Substance use	No	50 (66.7)	21 (28.0)	< 0.001*	
	Yes	25 (33.3)	54 (72.0)		
**Psychiatry**					
Mood Disorder (i.e. MDD)	No	73 (97.3)	69 (92.0)	0.276	
	Yes	2 (2.7)	6 (8.0)		
Anxiety (i.e. GAD, phobias)	No	73 (97.3)	74 (98.7)	1.000	
	Yes	2 (2.7)	1 (1.3)		
Schizophrenia/Psychosis	No	73 (97.3)	61 (81.3)	0.003*	
	Yes	2 ( 2.7)	14 (18.7)		
PTSD	No	75 (100)	75 (96.0)	0.245	
	Yes	0	3 (4.0)		
Bipolar I Disorder	No	75 (100%)	73 (97.3)	0.497	
	Yes	0	2 (2.7)		
Major neurocognitive disorder (i.e. dementia)	No	75 (100%)	75 (100%)		
Any Psychiatry comorbidity	No	70 (93.3)	50 (66.7)	< 0.001*	
	Yes	5 (6.7)	25 (33.3)		

* The asterisk and bold in* p-*value both meant to identify the statistically significant differences

Abbreviations: BMI: Body mass index, SNF: Skilled nursing facility, LTAC: Long term acute care, HTN: Hypertension, CKD: Chronic kidney disease, ESRD: End-stage renal disease, COPD: Chronic obstructive pulmonary disease, HIV/AIDS: Human immunodeficiency virus/Acquired immunodeficiency syndrome, MDD: Major depressive disorder, GAD: Generalized anxiety disorder, PTSD: Post-traumatic stress disorder

There was no difference in left ventricular end diastolic volume indexed between the MAHF (103 ml/m2, 79-130 ml/m2) or HF control groups (94 ml/m2, 73-118 ml/m2). Additionally, there was no difference in left ventricular systolic function based on left ventricular ejection fraction, between the MAHF (28%, 20–36%) and HF control (25%, 19–35%) groups. Furthermore, there were no differences in left atrial size, right ventricular size, or function, presence of ventricular thrombi, or pulmonary artery pressure in patients with MAHF compared to controls (Table 2). There was an effective sample size of 54 patients who had appropriate diastolic parameters for measurement (25 controls, 29 cases); we did not identify significant differences in diastolic function between the two groups.

**Table 2:** Transthoracic echocardiogram characteristics among cases with heart failure due to methamphetamine use compared to controls with heart failure due to other etiologies (*n* = 150)

		HF Control (*N* = 75)	MAHF (*N* = 75)	*p*-value
Biplane EF (%)	Median (IQR)	25.0 (19–35 )	28.0 (20–36 )	0.369
LVEDVi (ml/m2)	Median (IQR)	93.6 (73–118)	103 (79–130)	0.082
LAV (MOD-bp) Index (ml/m2)	Median (IQR)	43.5 (37–54 )	44.8 (35–54 )	0.907
RVDd (cm)	Median (IQR)	4.4 (4.0 -4.8)	4.5 (4.1-5.0)	0.229
RV TAPSE (cm)	Median (IQR)	1.6 (1.3–1.9)	1.6 (1.3–1.8)	0.687
RV S’ (cm/s)	Median (IQR)	9.1 (8.1–11.7)	9.9 (8.4–12.1)	0.534
RV FAC (%)	Mean (STD)	21.5 (6.42)	23.7 (7.85)	0.375
E/A	Median (IQR)	2.1 (1.8–3.1)	1.4 (0.9–2.1)	0.002*
LV diastolic function	Normal	1 (4.0)	0	0.067
	Grade I - impaired LV relaxation	3 (12.0)	7 (24.1)	
	Grade 2 - evidence of elevated filling pressures (in addition to impaired relaxation)	5 (20.0)	12 (41.4)	
	Grade 3 - severely elevated left sided filling pressure (restrictive pattern)	16 (64.0)	10 (34.5)	
RV Size	Normal	31 (41.3)	25 (33.3)	0.735
	Mildly dialiated	19 (25.3)	23 (30.7)	
	Moderately dialated	17 (22.7)	17 (22.7)	
	Severely Dialated	7 (9.3)	9 (12.0)	
RV systolic function	Normal	35 (46.7)	25 (33.3)	0.169
	Mild dysfunction	12 (16.0)	21 (28.0)	
	Moderate dysfunction	12 (16.0)	15 (20.0)	
	Severe dysfunction	15 (20.0)	11 (14.7)	
Ventricular thrombus?	None	65 (86.7)	63 (84.0)	0.685
	LV	7 (9.3)	8 (10.7)	
	RV	0	2 (2.7)	
	Both	1 (1.3)	2 (2.7)	
Moderate or severe TR?	No	58 (78.4)	54 (72.0)	0.368
	Yes	16 (21.6)	21 (28.0)	
Moderate or severe MR?	No	56 (74.7)	61 (81.3)	0.839
	Yes	14 (18.7)	14 (18.7)	
Moderate or severe AR?	No	70 (93.3)	70 (93.3)	0.620
	Yes	1 (1.3)	3 (4.0)	
RA Pressure (mmHg)	Low (0–5)	20 (29.4)	22 (33.8)	0.152
	Normal (5–10)	14 (20.6)	18 (27.7)	
	Mildly elevated (10–15)	17 (25.0)	7 (10.8)	
	Moderately elevated (15–20)	17 (25.0)	16 (24.6)	
	Severely elevated (> 20)	0	2 (3.1)	
PASP (mmHg)	Normal ( < = 35)	23 (41.1)	16 (29.6)	0.655
	Mildly elevated (36–45)	14 (25.0)	17 (31.5)	
	Moderate (46–64)	17 (30.3)	18 (33.3)	
	Severe ( > = 65)	2 (3.6)	3 (5.6)	

*p-values are uncorrected, and Bonferroni threshold is 0.0029

Abbreviations: IQR: Interquartile Range, LVEDVi: Left Ventricular End-Diastolic Volume Index, LAV: Left Atrial Volume, RV S’: Right Ventricle Systolic Excursion Velocity, RVDd: Right Ventricular Diastolic Dimension, TAPSE: Tricuspid Annular Plane Systolic Excursion, FAC: Functional Area Change, PASP: Pulmonary Artery Systolic Pressure

## Discussion

In this matched cohort, patients with MAHF had echocardiographic findings indistinguishable from HF controls at the time of heart failure diagnosis. This study adds to prior data on the echocardiographic characteristics of MAHF by focusing on echocardiograms from the time of initial diagnosis. Identification of echo parameters that can differentiate MAHF from other causes of HF would hold therapeutic value by allowing for early initiation of disease modifying therapy with interventions for methamphetamine cessation and heart failure GDMT prior to the development of myocardial fibrosis. Prior echocardiographic studies of chronic MAHF reported larger left atria, larger left ventricular end-diastolic volumes, lower LV ejection fractions, more severe RV dysfunction, and higher prevalence of LV thrombi when compared to patients with non-MAHF [4, 5]. In comparison to these studies of chronic MAHF, our cohort did not demonstrate significant differences in LV thrombi or other echocardiographic parameters. We speculate that these discrepancies are due to capturing a unique phenotype of MAHF in this cohort: earlier-stage disease with possibly shorter duration of methamphetamine exposure, less associated myocardial fibrosis along with less ventricular remodeling. As we did not collect data on the duration, frequency, or cumulative dose of methamphetamine use, this explanation remains speculative and requires prospective validation with detailed exposure histories. Of note, as this was the sentinel hospitalization for both MAHF patients and controls with heart-failure, it is unlikely that differential exposure to GDMT at the time of their first TTE impacted our findings. Notably, housing instability, public insurance, and psychiatric comorbidity are well-described features of populations who use methamphetamine and are not unique to this cohort [6]. Nonetheless, these social drivers of health may have influenced access to care, continuity of follow-up, or timing of clinical presentation in ways not captured by this cross-sectional comparison and warrant further investigation.

The findings from this study have important clinical implications when assessing a patient with suspected MAHF. Diagnostically, when MAHF is suspected in an individual with new-onset heart failure, clinicians should rely on multiple forms of data (clinical history and UDS), rather than specific imaging features to identify and diagnose MAHF. Furthermore, if MAHF is indistinguishable by echo from other forms of HF, patients who use methamphetamine and develop heart failure warrant a thorough work-up to exclude other forms of reversible cardiomyopathies. Prior work suggests that patients with MAHF do not get a comprehensive evaluation for alternative etiologies of heart failure, despite MAHF being a diagnosis of exclusion [7]. Contrasting these negative findings with prior studies demonstrating more severe biventricular dysfunction and remodeling suggests that MAHF has a more malignant clinical course from the time of diagnosis to the time of follow-up imaging, perhaps due to inequities in heart failure therapy or methamphetamine being such a highly cardiotoxic substance [8].

Based on the knowledge that methamphetamine cessation and heart failure GDMT lead to improved left ventricular geometry and function, future echocardiographic studies of MAHF should investigate the role of global longitudinal strain (GLS) in detecting subclinical heart failure in people who use methamphetamine. GLS is already widely used in cardio-oncology and transplant cardiology and could be similarly applied to other toxin-associated cardiomyopathies [9, 10]. We hope that differences in strain analyses may allow for early identification and intervention on this highly morbid condition.

This study is not without limitations, as the retrospective design of this single center study limits its generalizability. Furthermore, the small sample size (*n* = 75) may limit our ability to detect subtle differences in echocardiographic parameters. Additionally, 64% of patients had incomplete diastolic parameters due to missing data, uninterpretable data in the setting of atrial fibrillation, or contradictory diastolic parameters. The study was underpowered to perform subgroup analyses based on the demographic differences noted between the two groups (housing instability, psychiatric diagnoses, BMI). While we speculated that patients had earlier stages of heart failure given this was their first hospitalization with heart failure, we did not have the data necessary to statistically assess differences in NYHA classes between cases and controls. Furthermore, our data relied on both UDS results and patient history regarding substance use, with underreporting suspected of the latter. The duration, frequency, and dose of methamphetamine use was not recorded or used for these analyses. As an initial analysis, our data also did not include follow-up results or subsequent clinical outcomes, which can be explored in future work.

## Data Availability

All data supporting the findings of this study are available upon request.
